# Microcirculation-guided resuscitation in sepsis: the next frontier?

**DOI:** 10.3389/fmed.2023.1212321

**Published:** 2023-07-05

**Authors:** Elisa Damiani, Andrea Carsetti, Erika Casarotta, Roberta Domizi, Claudia Scorcella, Abele Donati, Erica Adrario

**Affiliations:** ^1^Anesthesia and Intensive Care Unit, Azienda Ospedaliero Universitaria delle Marche, Ancona, Italy; ^2^Department of Biomedical Sciences and Public Health, Università Politecnica delle Marche, Ancona, Italy

**Keywords:** sepsis, septic shock, microcirculation, sublingual videomicroscopy, tissue perfusion, glycocalyx

## Abstract

Microcirculatory dysfunction plays a key role in the pathogenesis of tissue dysoxia and organ failure in sepsis. Sublingual videomicroscopy techniques enable the real-time non-invasive assessment of microvascular blood flow. Alterations in sublingual microvascular perfusion were detected during sepsis and are associated with poor outcome. More importantly, sublingual videomicroscopy allowed to explore the effects of commonly applied resuscitative treatments in septic shock, such as fluids, vasopressors and inotropes, and showed that the optimization of macro-hemodynamic parameters may not be accompanied by an improvement in microvascular perfusion. This loss of “hemodynamic coherence,” i.e., the concordance between the response of the macrocirculation and the microcirculation, advocates for the integration of microvascular monitoring in the management of septic patients. Nonetheless, important barriers remain for a widespread use of sublingual videomicroscopy in the clinical practice. In this review, we discuss the actual limitations of this technique and future developments that may allow an easier and faster evaluation of the microcirculation at the bedside, and propose a role for sublingual microvascular monitoring in guiding and titrating resuscitative therapies in sepsis.

## 1. Introduction

Microcirculatory dysfunction plays a key role in the pathogenesis of tissue dysoxia and organ failure in sepsis (1). The ultimate goal of resuscitation in septic shock must be the optimization of microvascular blood flow. Systemic hemodynamic parameters, such as cardiac output (CO) and arterial pressure (AP), mixed venous O_2_ saturation (SvO_2_) or lactate, are usually applied as surrogates of tissue perfusion. Nonetheless, shock is often characterized by a loss of “hemodynamic coherence,” i.e., the concordance between the responses of the macrocirculation and the microcirculation (2): a therapeutic approach that purely targets macro-hemodynamics may thus pose the patients at risk for over- or under-resuscitation. Although a microcirculation-guided approach to the resuscitation of septic patients is desirable (3), important barriers remain for the application of microvascular monitoring alongside the standard hemodynamic parameters.

In this review, we describe the potential role of sublingual videomicroscopy for monitoring the microcirculation and guiding therapy during sepsis, and focus on the obstacles to its widespread use in the daily clinical practice.

## 2. Microcirculatory dysfunction during sepsis

Microvascular perfusion is regulated by a complex interplay of neuroendocrine, paracrine and mechanosensory pathways that adapt the local O_2_ supply to metabolic needs. These mechanisms are compromised during sepsis, as the inflammatory cascade and oxidative stress lead to endothelial dysfunction (4). The nitric oxide (NO) pathway is severely disturbed with heterogeneous over-expression of the inducible NO synthase and pathological shunting of blood flow in the microcirculation (1). Capillary hemorheology is altered due to a loss of red blood cell (RBC) deformability and tendency to aggregation (4). The endothelial glycocalyx is disrupted, causing capillary leakage, tissue oedema, coagulation abnormalities and enhanced leukocyte-endothelium interaction (5). Heterogeneity in capillary blood flow distribution hampers tissue O_2_ extraction, since shunted hypoxic areas occur next to normally or hyper-perfused areas despite preserved total blood flow (6).

The evaluation of the microcirculation has long been limited to the pre-clinical setting (7–9) due to the lack of technologies applicable at the bedside. In 1999, orthogonal polarization spectral (OPS) imaging was introduced and incorporated in a handheld videomicroscope, allowing a non-invasive *in vivo* observation of flowing RBCs in microvascular beds covered by thin epithelial layers, such as mucosal surfaces (10). De Backer et al. were the first to use OPS in patients with severe sepsis or septic shock and describe a reduction in sublingual microvascular density and the presence of vessels with intermittent or stopped flow (11). Most importantly, these alterations were more severe in non-survivors (11). In the following 20 years, a second generation device, termed sidestream dark field (SDF) (12), and recently a third generation videomicroscope called incident dark field (IDF) (13) were introduced and enabled to obtain images of progressively higher quality, sharper resolution and improved magnification.

Multiple studies used sublingual videomicroscopy to explore sepsis-induced microvascular abnormalities and their relationship with outcome (14–17). Sakr et al. (15) demonstrated that microcirculatory alterations improved rapidly in septic shock survivors, whereas no improvement was observed in patients dying with multiple organ failure, regardless of whether shock had resolved. De Backer et al. (17) found no correlation between the percentage of perfused vessels in the microcirculation and systemic hemodynamic parameters, and microcirculatory alterations were the strongest independent predictors of outcome. Sublingual videomicroscopy also enables to estimate the status of the endothelial glycocalyx by measuring the Perfused Boundary Region (PBR), i.e., the dimension of the permeable part of the glycocalyx allowing the penetration of RBCs (18). The sublingual PBR tended to be higher in septic patients as compared to healthy volunteers or non-septic critically ill patients (18), and a higher PBR was associated with worse outcome (19). The PBR was correlated with the number of rolling leukocytes in the microcirculation, supporting the role of glycocalyx shedding in enhancing leukocyte-endothelium interactions (18). A higher number of adhered leukocytes was found in the sublingual microcirculation of sepsis non-survivors (20).

## 3. How to resuscitate the microcirculation in sepsis?

Resuscitation strategies generally aim to normalize global hemodynamic parameters, with the expectation that this will result in a parallel improvement of tissue perfusion and oxygenation in vital organs. However, this may not happen in cases of sepsis/septic shock in which the “hemodynamic coherence” is lost.

The amount of fluids administered was not correlated with changes in microvascular vessel density during septic shock, whereas the relationship between capillary recruitment and fluid dose was preserved in cardiac surgery patients (21). Fluid administration was able to improve microvascular perfusion within the first 24 h of sepsis but not in a later phase, and the effect was independent of global hemodynamic changes (22). In preload-responsive septic patients, non-linear relationships were found between changes in CO and changes in microvascular perfusion in response to a passive leg raising or a volume expansion, suggesting that different mechanisms are implicated in the macro- and micro-vascular responses (23). In a general ICU population, a fluid challenge improved microcirculatory perfusion only in patients with abnormal microvascular blood flow at baseline, while no effect was observed in those with no significant alterations (24). Again, the microvascular response was independent of changes in stroke volume (24).

Similarly, the microvascular response to vasoactive agents is not straightforward (25). In general, those patients with more severe microvascular alterations are the ones who show a greater improvement in microcirculatory perfusion after the administration of vasoactive medications such as norepinephrine (26) or dobutamine (27), while the mere optimization of macro-hemodynamic parameters (e.g., mean arterial pressure [MAP] or CO) is not sufficient to guarantee a better microvascular blood flow (28). In hypertensive patients, titrating norepinephrine dose to increase MAP from 65 mmHg to usual levels resulted in improved microvascular density and flow (29). In septic shock, the calcium-sensitizer levosimendan improved microvascular perfusion better than 5 mcg/kg*min dobutamine, despite similar macro-hemodynamic effects (30).

As regards blood transfusions, again microvascular perfusion seemed to improve only when significant alterations were present at baseline (31, 32). The type of transfused blood is also important. The transfusion of leukodepleted RBCs might have a favorable effect on microcirculatory flow in sepsis (32). Conversely, the transfusion of old blood (with prolonged storage) may induce an increase in plasma free Hb, which acts as vasoconstrictor (33).

## 4. Barriers to the implementation of microvascular monitoring in sepsis

Significant barriers remain for the integration of sublingual microvascular monitoring in the management of septic patients:

- Difficulties in image acquisition and analysis;
- Concerns regarding the reliability of the sublingual mucosa as a window to the microcirculation of vital organs;
- Lack of well-defined targets among microvascular variables;
- Lack of specific therapies for “micro-hemodynamic” resuscitation.

### 4.1. Issues related to image acquisition and analysis

Low-quality videos may produce spurious microcirculatory data: inadequate focus/contrast or pressure artifacts prevent the visualization of some blood vessels, introducing a substantial bias in microvascular assessment. In a large database of videos recorded from critically ill patients, more than 50% were of unacceptable quality (34). Low-quality videos yielded worse microvascular parameters, (falsely) indicating lower vessel densities and worse perfusion quality (34). Ensuring artifact-free videos is a prerequisite for a reliable microcirculatory evaluation (35). Image quality should be systematically checked (36) and low-quality videos excluded. Technological developments of videomicroscope devices, such as the introduction of the lightweight easy-to-handle Cytocam (13), have increased their ease of use. A 415 nm blue light probe was recently tested instead of the commonly used 520 nm green light probe and was able to obtain clearer microcirculatory images, providing higher image quality and higher vessel densities (37).

The time-consuming offline analysis is another criticism. Numerous attempts have been made to obtain a faster point-of-care microcirculatory assessment. Tanaka et al. showed good agreement between a real-time qualitative bedside evaluation of the microcirculation made by nurses and the conventional offline analysis (38). Similarly, a rapid subjective categorization of microcirculation as “good,” “bad,” “very bad” may be sensitive and specific enough to identify the presence of microvascular abnormalities (39). Watchorn et al. applied the Point-of-care Microcirculation tool (POEM) score, a five-point score based on visual assessment of overall microcirculatory flow, showing good reproducibility (40).

Newly developed automated software systems were recently introduced, allowing an instantaneous quantitative bedside microcirculatory assessment, although their accuracy did not always prove optimal (41–44). In 2019, Hilty et al. (45) validated a novel algorithm in the MicroTools software package for the automated analysis of videomicroscopy image sequences, enabling for the first time an objective measurement of the absolute RBC velocities and capillary density. By using this algorithm, they were able to identify alterations associated with diseases and mechanisms of resuscitation in a wide range of perioperative and critically ill patient populations, laying the ground for the point-of-care application of microcirculation monitoring in the clinical setting (46).

### 4.2. Is the sublingual region a reliable window to the microcirculation of inner organs?

The sublingual mucosa is an ideal site for hand-held videomicroscopy for its easy accessibility and rich vascularization. Moreover, the tongue shares the same embryologic origin as the intestine: this is an interesting aspect, given the crucial role of the gut in the pathogenesis of multiple organ failure (47). However, studies exploring other microvascular beds showed possible discrepancies in some conditions. In patients with abdominal sepsis and a newly constructed intestinal stoma, no correlation was found between the sublingual and intestinal microcirculation at day 1; at day 3, the concordance between the two compartments was restored, probably due however to a normalization of perfusion in both regions (48). In a model of hyperdynamic septic shock due to cholangitis without changes in intra-abdominal pressure, microcirculatory alterations were similar in the sublingual and intestinal sites (49). In a hypodynamic sepsis model, the correlation between the sublingual and other microvascular beds was time-dependent, disappearing 5 h after the induction of sepsis (50). Of note however, the sublingual microcirculation showed the most pronounced alterations, suggesting that monitoring this area could enable to identify or rule out even subtler abnormalities in other capillary beds (50). In post-operative patients with abdominal sepsis, the sublingual and intestinal mucosal microcirculation were dissociated both at baseline and after a fluid challenge (51). Moreover, non-survivors showed more severe alterations in the intestinal villi microcirculation but not sublingually (51).

From these data, we can draw the following conclusions. The presence of an altered sublingual microcirculation is always an indicator of a possible impairment in microvascular perfusion of inner organs, and is associated with worse outcome. Conversely, the absence of sublingual microcirculatory alterations does not rule out abnormalities in other compartments; similarly, we could observe a “normalization” in the sublingual blood flow in response to therapy while perfusion remains suboptimal in other sites. The discrepancy may be more pronounced in some patient categories (e.g., patients with abdominal sepsis). These points do not question the potential utility of the sublingual microcirculation as a surrogate for microvascular perfusion of inner organs, keeping in mind that each microvascular bed has its own anatomical and physiological peculiarities and may be affected differently by the septic process. As a matter of fact, other hemodynamic parameters such as MAP or CO are not themselves absolute indicators of perfusion pressure or blood flow in specific organs, although commonly used as targets for therapy.

### 4.3. Lack of well-defined microcirculatory targets

Data on the clinical relevance of microvascular alterations have been predominantly expressed so far in terms of percentage of perfused vessels (PPV) and microvascular flow index (MFI), two parameters that describe “convection” (35) and are easier to interpret and compare between patients. While an MFI <2.6 generally indicates clinically relevant blood flow alterations (35), absolute quantitative cut-offs for vessel density parameters (describing “diffusion”) are more difficult to define.

While “normal values” for the sublingual microcirculation have been described in healthy volunteers (52, 53), pre-existing comorbidities may influence the microcirculation in septic patients: chronic arterial hypertension (54) and diabetes (55) were both associated with lower vessel density. On the other hand, a higher microcirculatory density does not always mean “healthier.” Sublingual vessel densities appeared higher than normal in patients with SARS-CoV-2 pneumonia (56, 57), because of hypoxia-induced capillary recruitment. Conversely, arterial hyperoxia can induce vasoconstriction (58, 59). These potentially confounding factors should be taken into account in the interpretation of microvascular density.

### 4.4. Lack of specific therapies for “micro-hemodynamic” resuscitation

Multiple efforts have been made to identify therapies specifically directed to the optimization of microvascular blood flow. In order to provide a comprehensive summary of the existing evidences, we performed a systematic review of studies evaluating possible microcirculatory-targeted therapies for sepsis. Studies were identified by searching Medline (PubMed) from its inception (main search was conducted on May 20th, 2023). The keywords “microcirculation,” “microvascular,” “sepsis,” “septic*,” “vasodilat*,” “nitric oxide,” “coagulation,” “clotting,” “microthrombosis,” “activated protein C,” “antithrombin,” “thrombomodulin,” “anticoagulant,” “anti-oxidant,” “vitamin C,” “blood purification,” “extracorporeal cytokine removal,” “hemoadsorption,” “immunomodulation” were typed in various combinations using Boolean operators. Both preclinical and clinical investigations were considered. After exclusion of non-pertinent articles, a total of 97 studies was identified focusing on the microvascular effects of different vasodilators, vasoconstrictors, antithrombotic/anti-platelet agents, anti-oxidants, blood purification or other type of treatments (including immunomodulant therapies). The main characteristics and findings of the studies are reported in the Supplementary Material. The vast majority were preclinical investigations (*N* = 79, 81%). Clinical studies were either uncontrolled/non-randomized trials (*N* = 10) (60–69) or randomized controlled trials on relatively small patient populations (*N* = 8) (70–77). Numerous attempts have been made to manipulate the NO pathway. However, while the use of NO synthase inhibitors was even deleterious in some animal models (Supplementary Material), clinical studies using inhaled NO or NO donors showed no benefit on the microcirculation (70, 72, 73). A large-scale multicentre RCT is currently ongoing on the vasodilator prostacyclin-analog ilomedin (78). Multiple studies targeted the interaction between endothelial dysfunction and coagulopathy. Activated protein C was associated with an improvement in microvascular perfusion both in animal models and in the clinical setting (63–68). Nonetheless, it was withdrawn from the market for safety reasons. Microvascular improvements were seen with antithrombin or thrombomodulin in animal models (Supplementary Material), although clinical data are lacking. Extracorporeal cytokine removal (61), antioxidants (e.g., vitamin-C (62)) or immunomodulant therapies (IgM-enriched immunoglobulins (77)) could play a role in resuscitating and/or protecting the microcirculation during septic shock, however further studies are needed to confirm their benefits.

## 5. Discussion

Sublingual videomicroscopy is a promising technique for evaluating the microcirculation, i.e., the real interface between blood and cells. For any treatment to be effective in optimizing tissue O_2_ availability, it is necessary that microvascular perfusion improves together with macro-hemodynamics. We reviewed the limitations of sublingual microvascular monitoring and the obstacles to its application in the clinical practice, including the lack of specific therapies. Importantly, high-quality clinical trials proving the efficacy of microcirculatory-targeted resuscitation strategies in sepsis are lacking.

**Figure 1:**
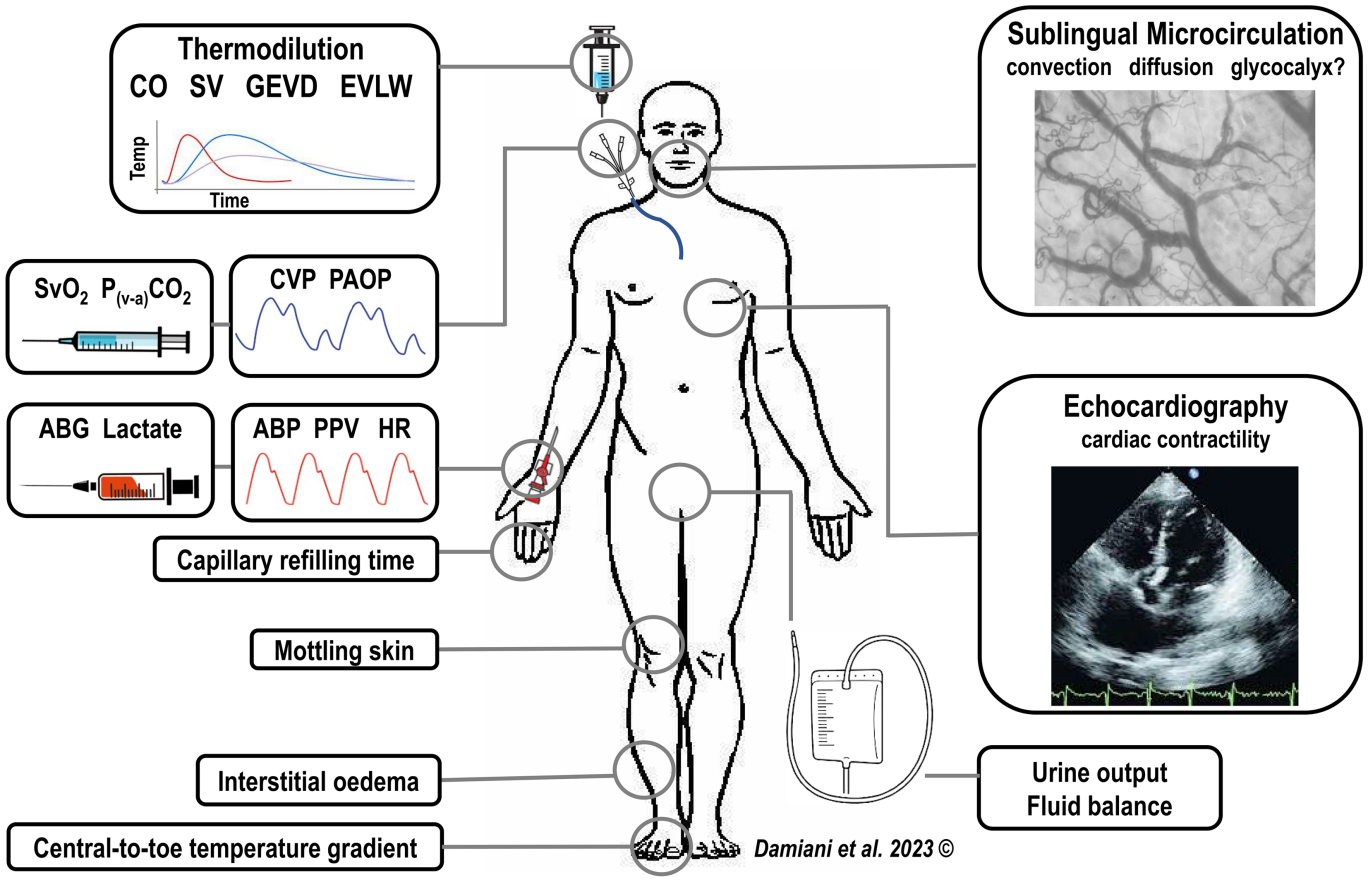
Integration of sublingual microcirculatory monitoring with a complete hemodynamic and physiological patient evaluation, including signs of tissue hypoperfusion (i.e., increased capillary refilling time, increased central-to-toe temperature difference, mottling skin) and the standard hemodynamic monitoring. CO, cardiac output, SV, stroke volume, GEDV, global end-diastolic volume, EVLW, extra-vascular lung water, CVP, central venous pressure, PAOP, pulmonary arterial occlusion pressure, ABG, arterial blood gasses, ABP, arterial blood pressure, PPV, pulse pressure variation, HR, heart rate.

Building a feasible and universally effective “microcirculation-centered” treatment algorithm for septic patients is difficult, given the high heterogeneity of sepsis as regards either the patients’ characteristics (e.g., underlying comorbidities), type of infection (e.g., abdominal versus pulmonary), severity of systemic inflammation and circulatory alterations varying over time in response to therapies. In such a complex scenario, the microcirculation could represent a tool for the personalization of therapy. Information coming from sublingual microcirculatory assessment should be part of a comprehensive hemodynamic and physiological monitoring and should be interpreted in the light of other markers of tissue perfusion (Figure 1).

**Figure 2:**
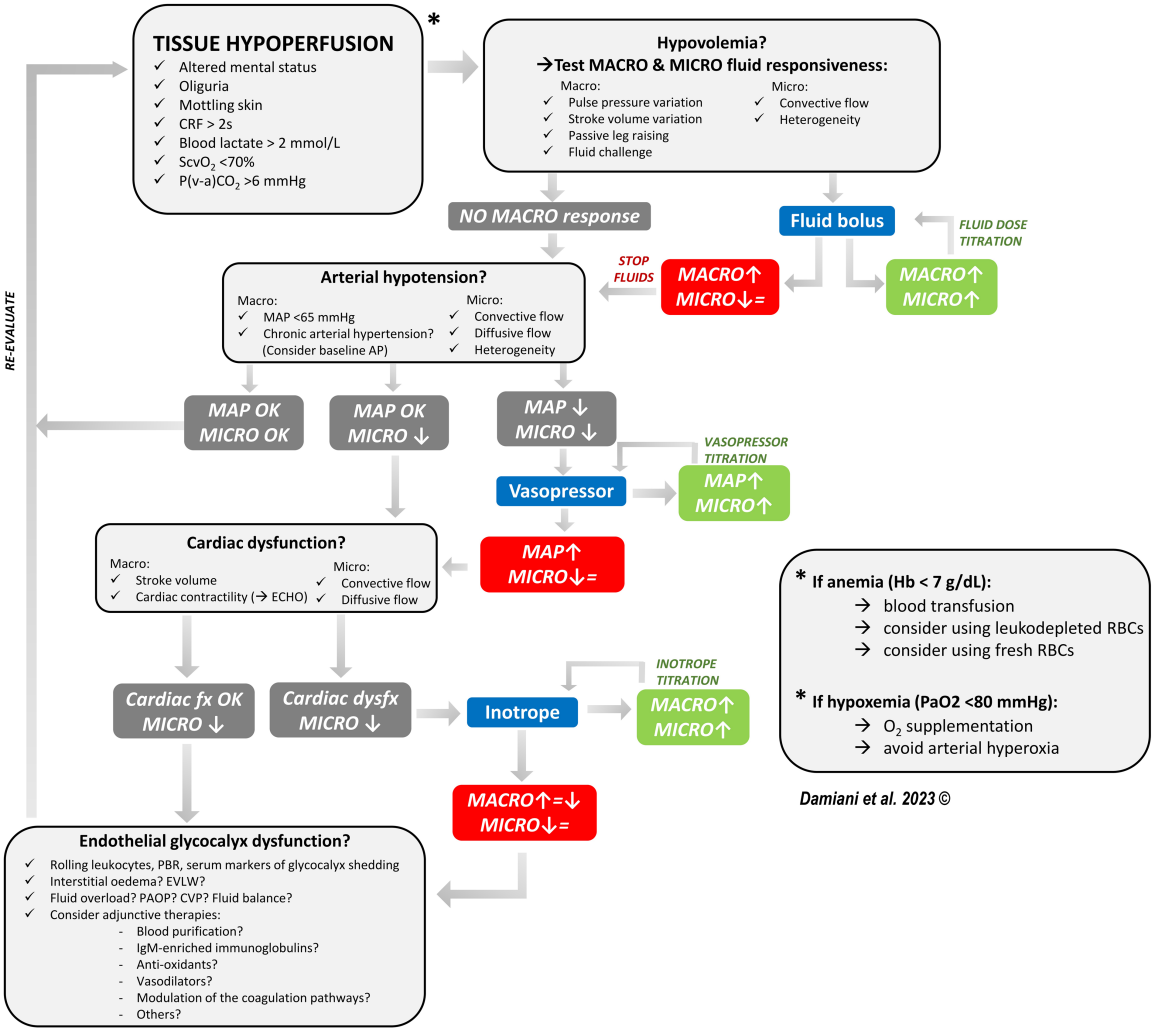
Proposed algorithm for the hemodynamic optimization of the septic patient, in which sublingual microvascular monitoring accompanies the standard hemodynamic monitoring and helps in guiding therapy and titrating fluid, vasopressor and inotrope dose. In presence of signs of tissue hypoperfusion, we should first ask if there is a condition of hypovolemia. Both macro- and micro-hemodynamic fluid responsiveness should be tested: if there is no sign of macro-hemodynamic fluid responsiveness, then fluids are not indicated. If there are signs of fluid responsiveness, a fluid bolus is indicated and both the macro-hemodynamic and the microcirculatory response should be assessed. Fluid infusion should be continued until there is an improvement in both macro-hemodynamics and the microcirculation (fluid titration). If macro-hemodynamics improves but the microcirculation does not (i.e., loss of hemodynamic coherence), this may be an indication to stop fluid infusion. Secondly, we should ask if there is significant vasodilation and the need for vasopressors. If MAP is too low and the microcirculation is altered, add a vasopressor. The vasopressor dose could be titrated until there is an improvement in microvascular perfusion following an increase in MAP (considering the patient’s baseline AP as a target). If a vasopressor-induced increase in MAP is not followed by an improved microvascular perfusion, this may be an indication to stop increasing the vasopressor dose. Thirdly, we should ask if there is cardiac dysfunction. If cardiac contractility is reduced, consider adding an inotrope. The inotrope dose could be titrated until there is an improvement in microvascular perfusion following an increase in the cardiac output. If the increase in cardiac output is not associated with an improvement in microvascular perfusion, this may be an indication to stop increasing the inotrope dose. If after optimizing volume status, vascular tone and cardiac contractility the microcirculation is still significantly altered, consider a possible derangement in the endothelial glycocalyx. The possible role of adjunctive therapies and other microcirculatory-targeted therapies should be evaluated in future studies. CRF capillary refilling time, MACRO macro-hemodynamic parameters, MICRO microcirculation, MAP mean arterial pressure, ECHO echocardiography, Hb hemoglobin, RBCs red blood cells, EVLW extravascular lung water, PAOP pulmonary arterial occlusion pressure, CVP central venous pressure, ↑ improvement, ↓ worsening, = stable.

In Figure 2, we propose a hemodynamic optimization algorithm for septic patients, in which microcirculatory monitoring plays a central role together with global hemodynamic parameters. This algorithm is based on three fundamental concepts.

First, we cannot rely purely on macro-hemodynamic targets when trying to restore tissue O_2_ delivery in sepsis, since the hemodynamic coherence may be lost. The classical treatments used for the hemodynamic optimization may fail to improve microvascular perfusion and even be deleterious. For example, fluid infusion induces hemodilution and decreases blood viscosity, potentially impairing shear stress-mediated regulation of vascular tone (2). High amounts of fluids in septic shock may cause iatrogenic endothelial injury and glycocalyx degradation (79). Previous studies showed that volume expansion, vasopressors, inotropes and even blood transfusions were able to recruit the microcirculation only if significant alterations were present at baseline, irrespective of the macro-hemodynamic response (24, 26, 27, 31, 32). Therefore, the evaluation of microvascular blood flow should accompany the standard hemodynamic monitoring.

Second, macro- and micro-circulatory responsiveness to treatments should be assessed simultaneously, in order to verify that any improvement in cardiac output or vascular tone results in increased capillary blood flow. Microvascular monitoring may help in guiding fluid, vasopressor and inotrope titration, in order to implement a patient-tailored therapy and avoid any over- or undertreatment. To this aim, it is imperative that technological developments of sublingual videomicroscopy allow an easier and fast assessment of microvascular parameters at the bedside, besides minimizing the possible biases due to poor image quality.

Third, we should consider the possible role of adjunctive therapies (e.g., extracorporeal blood purification) in optimizing microvascular hemorheology (62, 78) and potentially protecting the endothelium from iatrogenic injuries (79).

Bruno et al. very recently evaluated the impact of integrating microvascular monitoring in the therapeutic plan of patients with shock (80). The knowledge of the status of the microcirculation influenced the decision-making process for fluids and vasopressors in a significant number of cases, but had no impact on 30-day mortality (80). Several limitations must be acknowledged: the inclusion of patients with different types of shock (with potentially different microvascular abnormalities), mismatch between the announced and performed treatment changes after microcirculatory evaluation in a substantial number of cases, limitation of life-sustaining therapy in almost half of the patients on day 2, re-assessment of the microcirculation only at 24 h, almost 30% of videos were of unacceptable quality (80). Nonetheless, this was the first large randomized controlled trial on a microcirculation-driven treatment algorithm in patients with shock.

Future studies should be focused on septic shock, exploring the impact of integrating microvascular monitoring in the decision-making process and the efficacy of microcirculation-guided treatment algorithms. Further efforts are needed to find specific therapies for resuscitating the septic microcirculation. In the meantime however, the microcirculation should become part of our clinical reasoning.

## Author contributions

ED, AC, EC, RD, CS, AD, and EA: conceptualization. ED: writing – original draft preparation. AC, EC, RD, CS, AD, and EA: writing – review and editing. EA and AD: supervision. All authors contributed to the article and approved the submitted version.
