# Supplementary material for: Microcirculation-guided resuscitation in sepsis: the next frontier?
Source: Front Med (Lausanne). 2023 Jul 5;10:1212321. doi: 10.3389/fmed.2023.1212321 (PMC10354242; doi:10.3389/fmed.2023.1212321)
Supplement: Supplementary file 1 [file Table_1.DOCX]

**Supplementary Material**

**Table - List of studies that evaluated microcirculatory-targeted therapies.**

| **Type of intervention** | **Study** | **Design** | **Subjects, model** | **Study treatment** | **Microcirculatory target** | **Methods for microvascular assessment** | **Main findings** | **Hemodynamic coherence** |
| --- | --- | --- | --- | --- | --- | --- | --- | --- |
| *Vasodilators* | Boerma et al. (2010) [s1] | RCT | Septic patients (n=70) | Nitroglycerin | Vascular tone | SDF, sublingual microcirculation | No effect on sublingual microvascular blood flow | Yes (no effect on macro-hemodynamics) |
|  | de Miranda et al. (2015) [s2] | preclinical study | Hamsters, LPS injection | IV milrinone | Vascular tone | Intravistal microscopy, skin microcirculation | Reduction in arteriolar vasoconstriction, capillary perfusion deficits, and inflammatory response; increase in survival. | No (no correlation between MAP and functional capillary density) |
|  | Dumbarton et al. (2017) [s3] | Preclinical study | Rats, fecal peritonitis or LPS infusion | Tetrahydrobiopterin (nitric oxide synthase cofactor) | Leukocyte-endothelium interaction, vascular tone | Intravital microscopy, intestinal microcirculation | Decreased leukocyte-endothelial adhesion and improved functional capillary density in endotoxemia | No (no effect on macro-hemodynamics) |
|  | Flemming et al. (2014) [s4] | preclinical study | Sprangue-Dawley rats, colon ascendens stent peritonitis | Rolipram (Phosphodiesterase 4 inhibitor) | Endothelial function (barrier property) | Fluorescein isothiocyanate–albumin extravasation, intravital microscopy (mesentery) | Stabilization of microvascular barrier, improvement in microcirculatory flow, decrease in proinflammatory cytokines | Yes (stabilization of MAP in treated animals) |
|  | Grotowska et al. (2022) [s5] | preclinical study | Piglets (n=11), colon perforation | Inhaled nitric oxide + IV hydrocortisone | Vascular tone | SDF, sublingual microcirculation | No improvement in sublingual microcirculation | Yes (correlation between microvascular flow index and MAP) |
|  | He et al. (2012) [s6] | preclinical study | Sheeps (n=14), fecal peritonitis | tetrahydrobiopterin (a nitric oxide synthase cofactor) | Endothelial function | SDF, sublingual microcirculation | Attenuated impairment in sublingual microcirculation, better preserved gas exchange, renal flow and urine output, prolonged survival. | Yes (prevention of the decrease in cardiac index and MAP) |
|  | Hersch et al. (1998) [s7] | preclinical study | Sprangue-Dawly rats, CLP | Sodium nitroprusside (selective exposure of an exteriorized ileal segment) | Vascular tone | Intravital microscopy, intestinal microcirculation | Normalization of arteriolar diameters and blood flow | Not evaluated |
|  | Holthoff et al. (2012) [s8] | preclinical study | Mice, CLP | Resveratrol | Endothelial function by scavenging reactive nitrogen species | Intravital microscopy, renal microcirculation | Improvement of renal microcirculation and decrease in the levels of reactive nitrogen species in tubules, improvement of renal function | No (no change in MAP or HR) |
|  | Holthoff et al. (2013) [s9] | preclinical study | Mice, CLP | Rolipram (phosphodiesterase 4 inhibitor) | Endothelial function (permeability), renal microvascular blood flow | Intravital microscopy, renal microcirculation | Restoration of renal capillary perfusion and reduction of microvascular permeability | No (reduction in MAP and increase in HR with rolipram) |
|  | Krysztopik et al. (1996) [s10] | preclinical study | Rats, E. Coli injection | IV pentoxifylline | Renal microvascular blood flow (vascular tone) | Intravital microscopy, renal microcirculation | Improvement in renal blood flow due to pre- and postglomerular vasodilation | No (no effect on MAP or HR) |
|  | Luiking et al. (2020) [s11] | RCT | Patients with septic shock (n=18) | I-arginine infusion | Increase in whole body arginine and nitric oxide production | Gastric tonometry, skin Laser Doppler flowmetry | No improvement in local perfusion (gastric tonometry) or organ function | Yes (no effect on global hemodynamics) |
|  | Pranskunas et al. (2011) [s12] | Open-label study | Patients with severe sepsis or septic shock (n=14) | Magnesium Sulphate infusion | Vascular tone | SDF, sublingual microcirculation | No improvement in sublingual microcirculation | Yes (no effect on systemic hemodynamics) |
|  | Rutai et al. (2020) [s13] | preclinical study | Sprangue-Dawly rats, fecal peritonitis | EndothelinA-R antagonist ETR-p1/fl peptide and/or EndothelinB1-R agonist IRL-1620 | Vascular tone | Intravital microscopy, intestinal microcirculation | The combined ETA-R-ETB1-R-targeted therapy improved the intestinal microcirculation (by >41%), and reversed mitochondrial dysfunction | Yes (improvement in MAP) |
|  | Schmidt et al. (1996) [s14] | preclinical study | Rats, LPS infusion | Low-dose dopamine | Vascular tone | Intravital microscopy, intestinal microcirculation | Attenuation of the reduction in intestinal villus blood flow. | No (no effect on MAP or HR) |
|  | Siegemund et al. (2007) [s15] | preclinical study | Pigs, LPS infusion | 3-morpholino-sydnonimine (SIN-1) (NO-donor) | Vascular tone | Ileal tonometry | Improvement in the P(CO(2)) gap (measured by means of ileal tonometry), increase in serosal muP(O(2)) above shock levels, mucosal muP(O(2)) was resuscitated to baseline levels | Not evaluated |
|  | Steeb et al. (1992) [s16] | preclinical study | Rats, E. Coli bacteremia | IV pentoxifylline | Vascular tone | Intravital microscopy, intestinal microcirculation | Prevention of small-intestine vasoconstriction and preserved microvascular blood flow | No (no effect on systemic hemodynamics) |
|  | Trzeciak et al. (2014) [s17] | RCT | Patients with severe sepsis (n=50) | Inhaled nitric oxide | Vascular tone | SDF, sublingual microcirculation | No improvement in sublingual microcirculation | No correlation between change in MFI and lactate clearance |
|  | Van der Voort et al. (2015) [s18] | RCT | Severe sepsis and septic shock patients (n=90) | nitroglycerin, enoximone, dopamine and dexamethasone | Vascular tone | SDF, sublingual microcirculation | No faster reduction in organ failure as compared to standard therapy | Not assessed |
|  | Vellinga et al. (2015) [s19] | Open-label pilot study | Patients with septic shock and microcirculatory alterations (n=10) | Ketanserin | Vascular tone, anti-thrombotic and anti-inflammatory activity | SDF, sublingual microcirculation | Improvement in microcirculatory perfusion | No (decrease in MAP, no change in CI) |
|  | Wafa et al. (2015) [s20] | preclinical study | Lewis, rats, LPS injection | desmopressin | Vascular tone, leukocyte-endothelial interactions | Intravital microscopy, intestinal microcirculation | Improvement in the intestinal microcirculation, reduction in TNF-alpha levels and number of adhering luekocytes | Yes (recovery of MAP and HR) |
|  | Wang et al. (2022) [s21] | RCT | Septic patients (n=40) | Shenfu injection for 5 days | Vascular tone | SDF, sublingual microcirculation | Improvement in sublingual microcirculation, reduction in biomarkers of endothelial dysfunction. | Not assessed |
|  |  |  |  |  |  |  |  |  |
| *Vasoconstrictors* | Hessler et al. (2019) [s22] | preclinical study | Sprangue Dawly rats (n=40), CLP | Glipizide (ATP-sensitive K-channel inhibitor) | Vascular tone | Intravital microscopy, intestinal (villi) microcirculation | Increase in blood pressure with no change in the villi microcirculation | No (no change in microcirculation despite increase in MAP and decrease in CI) |
|  | Hwang et al. (2003) [s23] | preclinical study | Rats, CLP | NG-nitro-L-arginine-methylester hydrochloride (NOS inhibitor) | Vascular tone | Laser Doppler flowmetry, hepatic microcirculation | Aggravation of the deterioration in hepatic microcirculation | Not assessed |
|  | Hwang et al. (2003) bis [s24] | preclinical study | Sprangue Dawly rats, LPS injection | Different NOS inhibitors | Vascular tone | Laser Doppler flowmetry, hepatic microcirculation | Aminoguanidine prevented the hypotensive effect similarly to L-NAME, but it maintained cardiac output, stroke volume and hepatic microcirculation better | Yes |
|  | Jourdain et al. (1997) [s25] | preclinical study | Piglets, LPS infusion | N omega-nitro-L-arginine methyl ester (L-NAME) | Microvascular thrombosis | Histology, lung and kidney | Increased activation of intravascular coagulation | Not assessed |
|  | Matejovic et al. (2007) [s26] | preclinical study | Pigs (n=16), IV infusion of Pseudomonas Aeruginosa | L-N6-[1-iminoethyl]-lysine (iNOS inhibitor) + 4-hydroxy-2,2,6,6-tetramethylpiperidine-N-oxyl (neutralizator of superoxide) | Inhibition of free radical production | Laser Doppler flowmetry and tonometry, ileal microcirculation | Prevented hypotension, attenuated the deterioration in ileal mucosal microcirculation, prevented mucosal acidosis | Yes |
|  | Nantais et al. (2014) [s27] | preclinical study | Rats, LPS injection | Methylene blue | Vascular tone, leukocyte-endothelial interaction | Intravital microscopy, ileal microcirculation | Decrease in leukocyte adhesion and improvement of functional capillary density in the intestinal microcirculation | Yes |
|  | Pullamsetti et a. (2006) [s28] | preclinical study | Wistar rats (n=24), LPS infusion | Non-selective or selective inhibition of iNOS | Vascular tone and inhibition of free radical production | Spectrophotometry, ileal tissue oxygenation | Increase in blood pressure, reduced plasma nitrite and nitrate, no increase in lactate levels | Yes |
|  | Rehberg et al. (2012) [s29] | preclinical study | Sheeps, MRSA-induced pneumonia | Selective V(1a) agonist | Vascular tone, endothelial function, leukocyte-endothelium interaction | Immunohistochemical and histological analyses, lung | Reduction in neutrophil migration and plasma levels of nitric oxide, reduction in vascular leakage | Yes |
|  | Rosengarten et al. (2009) [s30] | preclinical study | Rats, LPS infusion | 1400W (specific iNOS inhibitor) | Vascular tone, inhibition of free radical production | Laser Doppler flowmetry, brain | No beneficial effects on the cerebral microcirculation | Yes |
|  |  |  |  |  |  |  |  |  |
| *Anti-thrombotics / anti-platelets* | Almac et al. (2013) [s31] | preclinal study | Wistar rats, LPS infusion | Recombinant human activated protein C | Inhibition of coagulation and inflammation | Phosphorimetry, renal microvascular oxygenation | No effect on renal microvascular oxygenation and function | Yes |
|  | Berthelsen et al. (2019) [s32] | RCT | Patients with septic shock (n=18) | Iloprost + eptifibatide | Platelet-endothelium interaction | Dosage of serum biomarkers of endothelial activation, platelet consumption and fibrinololysis | Reduction of endothelial injury, platelet consumption and fibrinolytic biomarkers along with improvement in SOFA score | Not assessed |
|  | Carestia et al. (2020) [s33] | preclinical study | Mice, IV or IP Staf. Aureus | Acetylsalicylic acid | Microvascular thrombosis, leukocyte-endothelium interaction and platelet aggregation | Intravital microscopy, liver microcirculation | Reduction in intravascular thrombin activity and microvascular occlusion | Not assessed |
|  | De Backer et al. (2006) [s34] | Prospective non-randomized study | Patients with severe sepsis (n=40) | Recombinant human activated protein C | Microvascular blood flow | OPS, sublingual microcirculation | Improvement in sepsis-induced microvascular alterations | No (no correlation between changes in microcirculation and MAP/CI) |
|  | Donati et al. (2009) [s35] | Prospective non-randomized study | Patients with severe sepsis or septic shock (n=16) | Recombinant human activated protein C | Microvascular reactivity and tissue oxygenation | Near Infrared Spectroscopy, skeletal muscle | Improvement in the skeletal muscle tissue oxygenation and microvascular reactivity | Yes (parallel improvement in tissue oxygenation and MAP) |
|  | Donati et al. (2013) [s36] | Prospective non-randomized study | Patients with severe sepsis (n=22) | Recombinant human activated protein C | Microvascular blood flow | SDF, sublingual microcirculation | Improvement in sublingual microvascular perfusion | No (no correlation between changes in microcirculation and changes in MAP/CI) |
|  | Favory et al. (2013) [s37] | prospective clinical study | Patients with severe sepsis (n=12) | Recombinant human activated protein C | Microvascular reactivity | Skin Laser Doppler | Improvement in macro- and microcirculatory vascular reactivity | Yes |
|  | Fischer et al. (2009) [s38] | preclinical study | Newborn piglets, LPS infusion | Recombinant human activated protein C | Microvascular blood flow | Intravital microscopy, intestinal microcirculation | Recover of functional capillary density and intestinal microcirculatory red blood cell velocity and red blood cell flow | Yes |
|  | Fuchs et al. (2010) [s39] | preclinical study | Lewis rats (n=40), colon ascendens stent peritonitis | Argatroban | Microvascular blood flow, leukocyte-endothelial interaction | Intravital microscopy, intestinal microcirculation | Improvement in intestinal microcirculation and reduction in leukocyte adherence | Not assessed |
|  | Hoffmann et al. (2004) [s40] | preclinical study | Hamsters, LPS injection | Recombinant human activated protein C | Leukocyte-endothelium interaction | Intravital microscopy, skin | Reduction in endothelial leukocyte adhesion and attenuation of microcirculatory dysfunction | Not assessed |
|  | Iba et al. (2002) [s41] | preclinical study | Wistar rats, LPS infusion | DX-9065a (Factor Xa inhibitor) | Leukocyte-endothelium interaction | Intravital microscopy, mesenteric microcirculation | Protective effect on the microcirculation by attenuating leukocyte-endothelial interaction | Not assessed |
|  | Iba et al. (2005) [s42] | preclinical study | Wistar rats, LPS infusion | Recombinant human activated protein C | Leukocyte-endothelium interaction | Intravital microscopy, mesenteric microcirculation | Inhibition of leukocyte-endothelial interaction and suppression of inflammatory cytokine production | Not assessed |
|  | Iba et al. (2005) bis [s43] | preclinical study | Wistar rats, LPS infusion | Antithrombin +daparinoid sodium or unfractionated heparin | Leukocyte-endothelium interaction | Intravital microscopy, mesenteric microcirculation | Suppression of leukocyte adhesion, red blood cell velocity better preserved with AT+DA | Not assessed |
|  | Iba et al. (2012) [s44] | preclinical study | Wistar rats, LPS infusion | Enoxaparin or fondaparinux | Endothelial function, Leukocyte-endothelium interaction | Intravital microscopy, mesenteric microcirculation | Reduction of leukocyte adherence to vascular endothelium and endothelial damage, with more prominent bleeding with fondaparinux | Not assessed |
|  | Iba et al. (2013) [s45] | preclinical study | Wistar rats, LPS infusion | Recombinant human thrombomodulin | Leukocyte-endothelium interaction | Intravital microscopy, mesenteric microcirculation | Suppression in leukocyte adhesion, thrombus formation and endothelial damage | Not assessed |
|  | Iba et al. (2014) [s46] | preclinical study | Wistar rats, LPS infusion | Antithrombin + recombinant human thrombomodulin | Leukocyte-endothelium interaction, microvascular blood flow | Intravital microscopy, mesenteric microcirculation | Suppression of leukocyte adhesion and thrombus formation, preservation of venular blood flow | Not assessed |
|  | Iba et al. (2015) [s47] | preclinical study | Wistar rats, LPS infusion | Recombinant human activated protein C | Leukocyte activation and endothelial function | Intravital microscopy, mesenteric microcirculation | Protective effects on the microcirculation, reduction in leukocyte cell death and netrophil extracellular traps formation | Not assessed |
|  | Iba et al. (2020) [s48] | preclinical study | Wistar rats, LPS injection | Recombinant antithrombin | Endothelial function and glycocalyx protection | Intravital microscopy, SDF (mesenteric microcirculation), serum biomarkers of glycocalyx injury | Maintenance of vascular integrity and microcirculation by preserving the glycocalyx | Not assessed |
|  | Kakino et al. (2022) [s49] | preclinical study | Mice, IP LPS | Recombinant human thrombomodulin | Glycocalyx protection | Fluorescence microscopy, electron microscopy, immunohistochemestry, heart | Protection of the capillary endothelium glycocalyx in the myocardium | Not assessed |
|  | Kato et al. (2021) [s50] | preclinical study | Mice, IP LPS | Recombinant human thrombomodulin | Thrombogenesis and endothelial function | Immunfluorescence, electron microscopy, lung and liver | Prevention of neutrophil extracellular traps formation in the organs and suppression of the increase in pro-inflammatory cytokines | Not assessed |
|  | Kawasaki et al. (2020) [s51] | preclinical study | Rats, sepsis models | Thrombomodulin alfa | Microvascular blood flow | non-contact optical imaging, skin microcirculation | Increase in microcirculatory blood velocity | Not assessed |
|  | Keller et al. (2011) [s52] | preclinical study | Rats, CLP | Recombinant human activated protein C | hepatic vasoactive gene and protein expression | Intravital microscopy, liver microcirculation | Improvement of hepatic perfusion index and red blood cell velocity | Not assessed |
|  | Kirschenbaum et al. (2006) [s53] | In vitro study | Plasma/serum from patients with septic shock | Recombinant human activated protein C and low-dose heparin | Neutrophil-platelet-endothelial cell interactions | Phase contrast microscopy, flowing cell suspensions on endothelial cells | rhAPC decreases sepsis-induced interactions between isolated platelets, neutrophils, and endothelial cells. Low-dose heparin attenuates the benefits observed with rhAPC. | Not assessed |
|  | Lehmann et al. (2006) [s54] | preclinical study | Lewis rats, LPS injection | Recombinant human activated protein C | Microvascular blood flow, leukocyte-endothelial interactions | Intravital microscopy, intestinal microcirculation | Increase in mucosal and muscular functional capillary density and reduction in the number of firmly adhering leucocytes in intestinal venules | No (no change in macrocirculation) |
|  | Lehmann et al. (2008) [s55] | preclinical study | Lewis rats, LPS injection | Recombinant human activated protein C | Leukocyte-endothelial interaction, endothelial function | Intravital microscopy, mesenteric microcirculation | Reduction in IL-1, leukocyte adherence and plasma extravasation in the mesenteric microcirculation | No (decrease in MAP) |
|  | Leithäuser et al. (2007) [s56] | preclinical study | Sprangue-Dawley rats, LPS infusion | Melagatran | Leukocyte-endothelial interaction, endothelial function | Intravital microscopy, mesenteric microcirculation | Prevention of the increase in microvascular permeability, leukocyte adherence and thrombin-antithrombin complex plasma concentration | Not assessed |
|  | Masip et al. (2013) [s57] | Observational study | Patients with severe sepsis or septic shock (n=58) | Recombinant human activated protein C | Microvascular blood flow and tissue oxygenation | Near Infrared Spectroscopy, skeletal muscle | Improvement in the skeletal muscle tissue oxygenation and microvascular reactivity | Yes (increase in MAP and reduction in norepinephrine dose) |
|  | Maybauer et al. (2010) [s58] | preclinical study | Sheeps, pulmonary instillation of live Pseudomonas Aeruginosa | Recombinant human activated protein C | Microvascular blood flow | Injection of coloured microspheres, several organs | Attenuation of the changes in visceral and cerebral microcirculation | Yes (stable cardiac output and HR, more stable MAP) |
|  | Piagnerelli et al. (2013) [s59] | Observational study | Patients with sepsis | Recombinant human activated protein C | Red blood cell deformability | ektacytometry | No clinically relevant effect on red blood cell deformability | Not assessed |
|  | Sorg et al. (2007) [s60] | preclinical study | Mice, LPS injection | Antithrombin | Prevention of microvascular thrombosis | Intravital microscopy, ear and cremaster muscle | Prevention of microvascular thrombotic occlusion | Not assessed |
|  | Suzuki et al (2020) [s61] | preclinical study | Mice, IP LPS | Recombinant human thrombomodulin | Preservation of endothelial glycocalyx | Histological, ultrastructural, and microarray analyses, lung microcirculation | Attenuation of inflammation and endothelial glycocalyx injuri in the pulmonary microcirculation | Not assessed |
|  | Wang et al. (2022) bis [s62] | preclinical study | Mice, LPS injection | Linagliptin (Dipeptidyl peptidase-4 inhibitor) | Anti-thrombotic effect | Fluorescence imaging, lung microcirculation | Linagliptin suppressed expressions of IL-1β and ICAM-1, attenuated TF expression, reduced thrombosis in the pulmonary microcirculation. | Not assessed |
|  | Zhang et al. (2021) [s63] | preclinical study | Beagle dogs, E. Coli infusion | Unfractionated heparin | Microvascular blood flow | SDF, intestinal microcirculation | Improvement in the small intestine microcirculatory perfusion | No |
|  | Zhou et al. (2012) [s64] | preclinical study | Lewis rats, LPS injection | Recombinant human activated protein C | Microvascular blood flow, leukocyte-endothelial interactions | Intravital microscopy, cerebral (pial) microcirculation | Improvement in the pial microcirculation by reducing leukocyte adhesion, increasing functional capillary density and reducing IL-1α levels | Not assessed |
|  |  |  |  |  |  |  |  |  |
| *Anti-oxidants* | Armour et al. (2001) [s65] | preclinical study | Sprangue-Dawley rats, CLP | IV ascorbate | Endothelial function | Intravital microscopy, skeletal muscle | Increase in perfused capillaries in the skeletal muscle microcirculation | Yes (prevention of MAP drop) |
|  | Baranutis et al. (2017) [s66] | In vitro study | Human lung endothelial cells, exposure to LPS | Hydrocortisone + vitamin C | Endothelial function | Assessment of transendothelial electrical resistance | Reverse of LPS-induced endothelial barrier dysfunction | Not assessed |
|  | Egan et al. (2001) [s67] | preclinical study | Sprangue-Dawley rats, LPS infusion | Taurine | Leukocyte-endothelium interactions | Intravital microscopy, mesenteric microcirculation | Decrease in leukocyte-endothelium interactions, adherence and transendothelial migration | Not assessed |
|  | Ergin et al. (2016) [s68] | preclinical study | Rats, LPS infusion | N-acetylcisteine | Modulation of inflammation and oxidative stress | Phosphorimetry, kidney | Improvement of cortical renal oxygenation, oxygen delivery, and oxygen consumption, reduction of the accumulation of NGAL or L-FABP, hyaluronic acid, and NO in the kidney | No (decrease in MAP) |
|  | Fisher et al. (2011) [s69] | preclinical study | Mice, LPS injection | IP ascorbate | Modulation of the proinflammatory, procoagulant state | Western blot analysis, lung | Attenuation of proinflammatory chemokine expression and microvascular thrombosis in the lungs | Not assessed |
|  | Guarda et al. (2015) [s70] | preclinical study | Wistar rats, IV E.Coli | Ethyl piruvate | Leukocyte-endothelial interactions | Intravital microscopy, mesenteric microcirculation | Reduction in the number of rolling, adherent and migrated leukocytes in the mesenteric microcirculation | Not assessed |
|  | Krysztopik et al. (1996) [s71] | preclinical study | Rats, live E. Coli injection | Lazaroid | Radical scavenging | Intravital microscopy, kidney | Attenuation of renal LPS-induced vasoconstriction and hypoperfusion | No (no increase in cardiac output) |
|  | Krysztopik et al. (1997) [s72] | preclinical study | Rats, live E. Coli injection | Lazaroid | Radical scavenging | Intravital microscopy, ileal microcirculation | Reduction of intestinal vasoconstriction and hypoperfusion | No (no increase in cardiac output) |
|  | Lavillegrand et al. (2022) [s73] | prospective non-randomized study | Patients with septic shock (n=30) | IV ascorbate | Antioxidant and anti-inflammatory properties | Iontophoresis, skin | Improvement in microvascular reactivity, mottling score, finger-tip and knee capillary refill time and central-to-peripery temperature gradient | No (no change in systemic hemodynamic parameter) |
|  | Matejovic et al. (2005) [s74] | preclinical study | Pigs, infusion of live Pseudomonas Aeruginosa | Tempol | Radical scavenging | Tonometry and Laser Doppler Flowmetry, ileal microcirculation | Attenuation of the deterioration in ileal mucosal microcirculation, reduction in sepsis-induced endothelial (von Willebrand factor) and hemostatic dysfunction (thrombin-antithrombin complexes, plasminogen activator inhibitor-type 1) | Yes |
|  | Maybauer et al. (2011) [s75] | preclinical study | Sheeps, Pseudomonas Aeruginosa-induced pneumonia | WW-85 (peroxynitrate decomposition catalyst) | Anti-oxidant activity | Western Blot, lung | Improvement in gas exchange and reduction in microvascular leakage | Not assessed |
|  | Secor et al. (2017) [s76] | In vitro study | Murine endothelial cells, exposure to LPS | Ascorbate pretreatment | Platelet-endothelial adhesion | Real-time PCR, immunofluorescence | Inhibition of platelet adhesion, reduced surface expression of P-selectin and vonWillebrand factor secretion | Not assessed |
|  | Tyml et al. (2005) [s77] | preclinical study | Sprangue-Dawley rats, CLP | IV ascorbate | Antioxidant and anti-inflammatory properties | Laser Doppler flowmetry, skeletal muscle | Prevention of the maldistribution of capillary blood flow in the skeletal muscle | Yes |
|  | Wang et al. (2023) [s78] | RCT | Patients with septic shock (n=22) | Hydrocortisone + vitamin C + vitamin B1 | Microvascular blood flow | SDF, sublingual microcirculation | Improvement in sublingual microcirculation at 4 and 24 hours after treatment | No (no change in MAP or cardiac output) |
|  | Wu et al. (2003) [s79] | preclinical study | Mice, CLP | IV ascorbate | Inhibition of iNOS expression | Western blot, real-time PCR, intravital microscopy, skeletal muscle | Decrease in oxidative stress, prevention of endothelial iNOS expression, and improvement of vascular reactivity in skeletal muscle | Yes (increase in MAP) |
|  | Wu et al. (2004) [s80] | preclinical study | Mice, CLP | IV ascorbate | Microvascular reactivity | Western blot, real-time PCR, intravital microscopy, skeletal muscle | Protection against the increase in plasma nitrite/nitrate concentration and against pressor response impairment | Not assessed |
|  | Zhou et al. (2012) [s81] | preclinical study | Mice, CLP | IV ascorbate | Endothelial function | Western blot, intravital microscopy, cremaster muscle | Protection against vascular leakage by inhibiting excessive production of NO and superoxide, formation of peroxynitrite, PP2A activation, and occludin dephosphorylation. | Not assessed |
|  |  |  |  |  |  |  |  |  |
| *Blood purification* | Sykora et al. (2009) [s82] | preclinical study | Pigs, fecal peritonitis | Coupled plasma filtration adsorption | Removal of circulation inflammatory mediators | SDF, ileal mucosa | No improvement in sepsis-induced alterations in microcirculation | Yes |
|  | Zuccari et al. (2020) [s83] | prospective observational study | Patients with sepsis/septic shock undergoing renal replacement therapy (n=9) | Cytosorb | Removal of circulation inflammatory mediators | SDF, sublingual microcirculation | Improvement in sublingual microcirculation | No (no variations in macro-hemodynamics) |
|  |  |  |  |  |  |  |  |  |
| *Others* | Alfieri et al. (2012) [s84] | preclinical study | Mice, LPS injection | MAT.Ang-1 (Matilin-1 Angiopoietin-1) | Modulation of inflammation, endothelial function | Intravital microscopy and Doppler fluximetry, skin microcirculation | Improved tissue perfusion, reduced inflammation | Not assessed |
|  | Domizi et al. (2019) [s85] | RCT | Patients with sepsis/septic shock (n=19) | IgM-enriched immunoglobulins | Modulation of inflammation, microvascular blood flow | SDF, sublingual microcirculation | Increase in sublingual microvascular perfusion | No (improvement in microcirculation independent of macro-hemodynamic changes) |
|  | Hoffman et al. (2008) [s86] | preclinical study | Hamsters, LPS injection | IgM-enriched immunoglobulins | Leukocyte-endothelial interaction, microvascular blood flow | Intravital microscopy, skin microcirculation | Reduction in leukocyte adhesion in venules, normalization of capillary perfusion at 24 hours | Not assessed |
|  | Kao et al. (2011) [s87] | preclinical study | Mice, fecal peritonitis | erythropoietin (rHuEPO) | Anti-inflammatory activity | Intravital microscopy, skeletal muscle | Improvement in capillary perfusion density due to the activation of eNOS | Not assessed |
|  | La Mura et al. (2013) [s88] | preclinical study | Rats, LPS injection | simvastatin | Microvascular blood flow, endothelial function | Western blot, PCR, histopathology | Prevention of the liver microvascular dysfunction (with treatment from 3 days before LPS) and reduced liver inflammation | Not assessed |
|  | Landsberger et al. (2010) [s89] | preclinical study | Lewis rats, LPS injection | Lectin-like oxidized low-density lipoprotein receptor-1 (LOX-1) antibodies | Leukocyte-endothelial interactions | Intravital microscopy, intestinal microcirculation | Reduction in leukocyte adhesion in intestinal submucosal venules | Not assessed |
|  | McCuskey et al. (1996) [s90] | preclinical study | Rats, CLP | Immunoglobulins G | Leukocyte-endothelial interaction, microvascular blood flow | Intravital microscopy, liver | Reduction of leukocyte-endothelial interactions in the liver | Not assessed |
|  | McGown et al. (2010) [s91] | preclinical study | Wistar rats (n=18), LPS infusion | Pravastatin | Modulation of inflammation | Intravital microscopy | Pravastatin preserved blood pressure, limited the increase in nitrite and the venular macromolecular leak, reduced leukocyte adhesion, increased endothelial cell expression of NOS III | Yes (improvement in MAP) |
|  | McGown et al. (2015) [s92] | preclinical study | Wistar rats (n=54), LPS infusion | Atorvastatin | Modulation of inflammation | Intravital microscopy | Decreased the upregulation of pro-inflammatory endothelial cell NOSII expression | Yes (improvement in MAP) |
|  | Reis et al. (2017) [s93] | preclinical study | Mice, fecal peritonitis | statins | Modulation of inflammation | Intravital microscopy, brain | Reverse of cerebral microcirculatory blood flow impairment | Not assessed |
|  | Stoyanoff et al. (2018) [s94] | preclinical study | Mice, LPS injection | erythropoietin | Endothelial function | Immunohistochemestry, Western blot analysis | Decreased expression of HIF-1alpha, iNOS, NF-kB; enhancement of vascular endothelium growth factor system | Not assessed |
|  | Ge et al. (2019) [s95] | Preclinical study | Rats, CLP | LDK378 (ALK-inhibitor) | Microvascular blood flow | SDF, sublingual microcirculation | Improvement of perfused small vessel density and microvascular flow index | Yes |
|  | Tang et al. (2023) [s96] | Preclinical study | Sprangue-Dawley rats, CLP | Dl-3-n-butylphthalide | Endothelial function (activation of PI3K/AKT signalling pathway, inhibition of autophagy) and microvascular blood flow | SDF, electron microscopy, intestinal microcirculation | Improvement in microvascular blood flow | No (no improvement in MAP or HR) |
|  | Damiani et al. (2016) [s97] | Preclinical study | Wistar rats, LPS infusion | 4% or 20% human serum albumin | Microvascular blood flow (by exerting anti-inflammatory and anti-oxidant activity) | SDF, skeletal muscle microcirculation | Improvement in microvascular blood flow, which was more stable with 20% human serum albumin | No (MAP was stable over time despite microvascular alterations) |

RCT randomized controlled trial, LPS lipopolysaccharide, CLP cecal ligation and puncture, SDF sidestream dark field, MAP mean arterial pressure, HR heart rate, CI cardiac index, OPS orthogonal polarization system

**References**

[s1] Boerma EC, Koopmans M, Konijn A, Kaiferova K, Bakker AJ, van Roon EN, et al. Effects of nitroglycerin on sublingual microcirculatory blood flow in patients with severe sepsis/septic shock after a strict resuscitation protocol: a double-blind randomized placebo controlled trial. Crit Care Med 2010, 38, 93-100.

[s2] De Miranda ML, Pereira SJ, Santos AO, Villela NR, Kraemer-Aguiar LG, Bouskela E. Milrinone attenuates arteriolar vasoconstriction and capillary perfusion deficits on endotoxemic hamsters. PLoS One 2015, 10, e0117004.

[s3] Dumbarton TC, Maxan A, Farah N, Sharawy N, Zhou J, Nantais J, et al. Tetrahydrobiopterin improves microcirculation in experimental sepsis. Clin Hemorheol Microcirc 2017, 67, 15-24.

[s4] Flemming S, Schlegel N, Wunder C, Meir M, Baar W, Wollborn J, et al. Phosphodiesterase 4 inhibition dose dependently stabilizes microvascular barrier functions and microcirculationin a rodent model of polymicrobial sepsis. Shock 2014, 41, 537-45.

[s5] Grotowska M, Harbut P, Frostell C, Goździk W. Fluid resuscitation, but not inhaled nitric oxide, improves microcirculation in septic pigs. Adv Clin Exp Med 2022, doi: 10.17219/acem/156700.

[s6] He X, Su F, Velissaris D, Salgado DR, de Souza Barros D, Lorent S, et al. Administration of tetrahydrobiopterin improves the microcirculation and outcome in an ovine model of septic shock. Crit Care Med 2012, 40, 2833-40.

[s7] Hersch M, Madorin WS, Sibbald WJ, Martin CM. Selective gut microcirculatory control (SGMC) in septic rats: a novel approach with a locally applied vasoactive drug. Shock 1998, 10, 292-7.

[s8] Holthoff JH, Wang Z, Seely KA, Gokden N, Mayeux PR. Resveratrol improves renal microcirculation, protects the tubular epithelium, and prolongs survival in a mouse model of sepsis-induced acute kidney injury. Kidney Int 2012, 81, 370-8.

[s9] Holthoff JH, Wang Z, Patil NK, Gokden N, Mayeux PR. Rolipram improves renal perfusion and function during sepsis in the mouse. J Pharmacol Exp Ther 2013, 347, 357-64.

[s10] Krysztopik RJ, Bentley FR, Wilson MA, Spain DA, Garrison RN. Vasomotor response to pentoxifylline mediates improved renal blood flow to bacteremia. J Surg Res 1996, 63, 17-22.

[s11] Luiking YC, Poeze M, Deutz NE. A randomized-controlled trial of arginine infusion in severe sepsis on microcirculation and metabolism. Clin Nutr 2020, 39, 1764-1773.

[s12] Pranskunas A, Vellinga NA, Pilvinis V, Koopmans M, Boerma EC. Microcirculatory changes during open label magnesium sulphate infusion in patients with severe sepsis and septic shock. BMC Anesthesiol 2011, 11:12.

[s13] Rutai A, Fejes R, Juhász L, Tallósy SP, Poles MZ, Földesi I, et al. Endothelin A and B Receptors: Potential Targets for Microcirculatory-Mitochondrial Therapy in Experimental Sepsis. Shock 2020, 54, 87-95.

[s14] Schmidt H, Secchi A, Wellmann R, Böhrer H, Bach A, Martin E. Effect of low-dose dopamine on intestinal villus microcirculation during normotensive endotoxaemia in rats. Br J Anaesth 1996, 76, 707-12.

[s15] Siegemund M, Van Bommel J, Sinaasappel M, Schwarte LA, Studer W, Girard T, et al. The NO donor SIN-1 improves intestinal-arterial P(CO(2)) gap in experimental endotoxemia: an animal study. Acta Anaesthesiol Scand 2007, 51, 693-700.

[s16] Steeb GD, Wilson MA, Garrison RN. Pentoxifylline preserves small-intestine microvascular blood flow during bacteremia. Surgery 1992, 112, 756-63.

[s17] Trzeciak S, Glaspey LJ, Dellinger RP, Durflinger P, Anderson K, Dezfulian C, et al. Randomized controlled trial of inhaled nitric oxide for the treatment of microcirculatory dysfunction in patients with sepsis. Crit Care Med 2014, 42, 2482-92.

[s18] Van der Voort PH, van Zanten M, Bosman RJ, van Stijn I, Wester JP, van Raalte R, et al. Testing a conceptual model on early opening of the microcirculation in severe sepsis and septic shock: a randomised controlled pilot study. Eur J Anaesthesiol 2015, 32, 189-98.

[s19] Vellinga NA, Veenstra G, Scorcella C, Koopmans M, van Roon EN, Ince C, et al. Effects of ketanserin on microcirculatory alterations in septic shock: An open-label pilot study. J Crit Care 2015, 30, 1156-62.

[s20] Wafa K, Lehmann C, Wagner L, Drzymulski I, Wegner A, Pavlovic D. Desmopressin improves intestinal functional capillary density and decreases leukocyte activation in experimental endotoxemia. Microvasc Res 2015, 97, 98-104.

[s21] Wang S, Liu G, Chen L, Xu X, Jia T, Zhu C, et al. Effects of shenfu injection on sublingual microcirculation in septic shock patients: a randomized controlled trial. Shock 2022, 58, 196-203.

[s22] Hessler M, Pinto BB, Arnemann PH, Kampmeier TG, Seidel L, Morelli A, et al. Differential Effects of Selective and Nonselective Potassium Channel Inhibitors in Ovine Endotoxemic Shock (Macrocirculation) and in a Rat Model of Septic Shock (Microcirculation). Shock 2019, 51, 247-255.

[s23] Hwang TL, Han ML. The changes of hepatic sinusoidal microcirculation and effects of nitric oxide synthase inhibitor during sepsis. Hepatogastroenterology 2003, 50, 213-6.

[s24] Hwang TL, Yeh CC. Hemodynamic and hepatic microcirculational changes in endotoxemic rats treated with different NOS inhibitors. Hepatogastroenterology 2003, 50, 188-91.

[s25] Jourdain M, Tournoys A, Leroy X, Mangalaboyi J, Fourrier F, Goudemand J, et al. Effects of N omega-nitro-L-arginine methyl ester on the endotoxin-induced disseminated intravascular coagulation in porcine septic shock. Crit Care Med 1997, 25, 452-9.

[s26] Matejovic M, Krouzecky A, Rokyta R Jr, Radej J, Kralova H, Treska V, et al. Effects of combining inducible nitric oxide synthase inhibitor and radical scavenger during porcine bacteremia. Shock 2007, 27, 61-8.

[s27] Nantais J, Dumbarton TC, Farah N, Maxan A, Zhou J, Minor S, et al. Impact of methylene blue in addition to norepinephrine on the intestinal microcirculation in experimental septic shock. Clin Hemorheol Microcirc 2014, 58, 97-105.

[s28] Pullamsetti SS, Maring D, Ghofrani HA, Mayer K, Weissmann N, Rosengarten B, et al. Effect of nitric oxide synthase (NOS) inhibition on macro- and microcirculation in a model of rat endotoxic shock. Thromb Haemost 2006, 95, 720-7.

[s29] Rehberg S, Yamamoto Y, Sousse L, Bartha E, Jonkam C, Hasselbach AK, et al. Selective V(1a) agonism attenuates vascular dysfunction and fluid accumulation in ovine severe sepsis. Am J Physiol Heart Circ Physiol 2012, 303, H1245-54.

[s30] Rosengarten B, Wolff S, Klatt S, Schermuly RT. Effects of inducible nitric oxide synthase inhibition or norepinephrine on the neurovascular coupling in an endotoxic rat shock model. Crit Care 2009, 13, R139.

[s31] Almac E, Johannes T, Bezemer R, Mik EG, Unertl KE, Groeneveld AB, et al. Activated protein C ameliorates impaired renal microvascular oxygenation and sodium reabsorption in endotoxemic rats. Intensive Care Med Exp 2013, 1, 24.

[s32] Berthelsen RE, Ostrowski SR, Bestle MH, Johansson PI. Co-administration of iloprost and eptifibatide in septic shock (CO-ILEPSS)-a randomised, controlled, double-blind investigator-initiated trial investigating safety and efficacy. Crit Care 2019, 23, 301.

[s33] Carestia A, Davis RP, Grosjean H, Lau MW, Jenne CN. Acetylsalicylic acid inhibits intravascular coagulation during Staphylococcus aureus-induced sepsis in mice. Blood 2020, 135, 1281-1286.

[s34] De Backer D, Verdant C, Chierego M, Koch M, Gullo A, Vincent JL. Effects of drotrecogin alfa activated on microcirculatory alterations in patients with severe sepsis. Crit Care Med 2006, 34, 1918-24.

[s35] Donati A, Romanelli M, Botticelli L, Valentini A, Gabbanelli V, Nataloni S, et al. Recombinant activated protein C treatment improves tissue perfusion and oxygenation in septic patients measured by near-infrared spectroscopy. Crit Care 2009, 13 Suppl 5, S12.

[s36] Donati A, Damiani E, Botticelli L, Adrario E, Lombrano MR, Domizi R, et al. The aPC treatment improves microcirculation in severe sepsis/septic shock syndrome. BMC Anesthesiol 2013, 13, 25.

[s37] Favory R, Poissy J, Alves I, Guerry MJ, Lemyze M, Parmentier-Decrucq E, et al. Activated protein C improves macrovascular and microvascular reactivity in human severe sepsis and septic shock. Shock 2013, 40, 512-8.

[s38] Fischer D, Nold MF, Nold-Petry CA, Furlan A, Veldman A. Protein C preserves microcirculation in a model of neonatal septic shock. Vasc Health Risk Manag 2009, 5, 775-81.

[s39] Fuchs C, Ladwig E, Zhou J, Pavlovic D, Behrend K, Whynot S, et al. Argatroban administration reduces leukocyte adhesion and improves capillary perfusion within the intestinal microcirculation in experimental sepsis. Thromb Haemost 2010, 104, 1022-8.

[s40] Hoffmann JN, Vollmar B, Laschke MW, Inthorn D, Fertmann J, Schildberg FW, et al. Microhemodynamic and cellular mechanisms of activated protein C action during endotoxemia. Crit Care Med 2004, 32, 1011-7.

[s41] Iba T, Kidokoro A, Fukunaga M, Fuse S, Suda M, Kunitada S, et al. Factor Xa-inhibitor (DX-9065a) modulates the leukocyte-endothelial cell interaction in endotoxemic rat. Shock 2002, 17, 159-62.

[s42] Iba T, Kidokoro A, Fukunaga M, Nagakari K, Shirahama A, et al. Activated protein C improves the visceral microcirculation by attenuating the leukocyte-endothelial interaction in a rat lipopolysaccharide model. Crit Care Med 2005, 33, 368-72.

[s43] Iba T, Kidokoro A, Fukunaga M, Nagakari K, Suda M, Yoshikawa S, et al. Antithrombin ameliorates endotoxin-induced organ dysfunction more efficiently when combined with danaparoid sodium than with unfractionated heparin. Intensive Care Med 2005, 31, 1101-8.

[s44] Iba T, Okamoto K, Ohike T, Tajirika T, Aihara K, Watanabe S, et al. Enoxaparin and fondaparinux attenuates endothelial damage in endotoxemic rats. J Trauma Acute Care Surg 2012, 72, 177-82.

[s45] Iba T, Aihara K, Watanabe S, Yanagawa Y, Takemoto M, Yamada A, et al. Recombinant thrombomodulin improves the visceral microcirculation by attenuating the leukocyte-endothelial interaction in a rat LPS model. Thromb Res 2013, 131, 295-9.

[s46] Iba T, Miki T, Hashiguchi N, Yamada A, Nagaoka I. Combination of antithrombin and recombinant thrombomodulin attenuates leukocyte-endothelial interaction and suppresses the increase of intrinsic damage-associated molecular patterns in endotoxemic rats. J Surg Res 2014, 187, 581-6.

[s47] Iba T, Nagakari K. The effect of plasma-derived activated protein C on leukocyte cell-death and vascular endothelial damage. Thromb Res 2015, 135, 963-9.

[s48] Iba T, Levy JH, Aihara K, Kadota K, Tanaka H, Sato K, et al. Newly Developed Recombinant Antithrombin Protects the Endothelial Glycocalyx in an Endotoxin-Induced Rat Model of Sepsis. Int J Mol Sci 2020, 22, 176.

[s49] Kakino Y, Doi T, Okada H, Suzuki K, Takada C, Tomita H, et al. Recombinant thrombomodulin may protect cardiac capillary endothelial glycocalyx through promoting Glypican-1 expression under experimental endotoxemia. Heliyon 2022, 8, e11262.

[s50] Kato Y, Nishida O, Kuriyama N, Nakamura T, Kawaji T, Onouchi T, et al. Effects of Thrombomodulin in Reducing Lethality and Suppressing Neutrophil Extracellular Trap Formation in the Lungs and Liver in a Lipopolysaccharide-Induced Murine Septic Shock Model. Int J Mol Sci 2021, 22, 4933.

[s51] Kawasaki M, Nakano K, Ohnishi T, Sekine M, Watanabe E, Oda S, et al. Investigation into Effect of Thrombomodulin Alfa for Septic Model Rats based on Microcirculation Image Analysis. Annu Int Conf IEEE Eng Med Biol Soc 2020, 2020, 1844-1847.

[s52] Keller SA, Moore CC, Clemens MG, McKillop IH, Huynh T. Activated protein C restores hepatic microcirculation during sepsis by modulating vasoregulator expression. Shock 2011, 36, 361-9.

[s53] Kirschenbaum LA, Lopez WC, Ohrum P, Tsen A, Khazin J, Astiz ME. Effect of recombinant activated protein C and low-dose heparin on neutrophil-endothelial cell interactions in septic shock. Crit Care Med 2006, 34, 2207-12.

[s54] Lehmann C, Meissner K, Knöck A, Diedrich S, Pavlovic D, Gründling M, et al. Activated protein C improves intestinal microcirculation in experimental endotoxaemia in the rat. Crit Care 2006, 10, R157.

[s55] Lehmann C, Scheibe R, Schade M, Meissner K, Gründling M, Usichenko T, et al. Effects of activated protein C on the mesenteric microcirculation and cytokine release during experimental endotoxemia. Can J Anaesth 2008, 55, 155-62.

[s56] Leithäuser B, Fassbender M, Eickhoff M, Elg M, Eichner G, Matthias FR. The direct thrombin inhibitor melagatran counteracts endotoxin-induced endothelial leukocyte adherence and microvascular leakage in the rat mesentery. Rationale for the treatment of inflammatory disorders beyond sepsis? Clin Hemorheol Microcirc 2007, 36, 277-89.

[s57] Masip J, Mesquida J, Luengo C, Gili G, Gomà G, Ferrer R, et al. Near-infrared spectroscopy StO2 monitoring to assess the therapeutic effect of drotrecogin alfa (activated) on microcirculation in patients with severe sepsis or septic shock. Ann Intensive Care 2013, 3, 30.

[s58] Maybauer MO, Maybauer DM, Fraser JF, Szabo C, Westphal M, Kiss L, et al. Recombinant human activated protein C attenuates cardiovascular and microcirculatory dysfunction in acute lung injury and septic shock. Crit Care 2010, 14, R217.

[s59] Piagnerelli M, Njimi H, Coelho TV, Reggiori G, Castanares Zapatero D, Donadello K, et al. Limited effects of activated protein C on red blood cell deformability. Clin Hemorheol Microcirc 2013, 53, 387-91.

[s60] Sorg H, Hoffmann JN, Rumbaut RE, Menger MD, Lindenblatt N, Vollmar B. Efficacy of antithrombin in the prevention of microvascular thrombosis during endotoxemia: an intravital microscopic study. Thromb Res 2007, 121, 241-8.

[s61] Suzuki K, Okada H, Takemura G, Takada C, Tomita H, Yano H, et al. Recombinant thrombomodulin protects against LPS-induced acute respiratory distress syndrome via preservation of pulmonary endothelial glycocalyx. Br J Pharmacol 2020, 177, 4021-4033.

[s62] Wang SC, Wang XY, Liu CT, Chou RH, Chen ZB, Huang PH, et al. The Dipeptidyl Peptidase-4 Inhibitor Linagliptin Ameliorates Endothelial Inflammation and Microvascular Thrombosis in a Sepsis Mouse Model. Int J Mol Sci 2022, 23, 3065.

[s63] Zhang H, Sun Y, An X, Ma X. Unfractionated Heparin Improves the Intestinal Microcirculation in a Canine Septic Shock Model. Mediators Inflamm 2021, 2021, 9985397.

[s64] Zhou J, Pavlovic D, Willecke J, Friedel C, Whynot S, Hung O, et al. Activated protein C improves pial microcirculation in experimental endotoxemia in rats. Microvasc Res 2012, 83, 276-80.

[s65] Armour J, Tyml K, Lidington D, Wilson JX. Ascorbate prevents microvascular dysfunction in the skeletal muscle of the septic rat. J Appl Physiol (1985) 2001, 90, 795-803.

[s66] Baranutis N, Khangoora V, Marik PE, Catravas JD. Hydrocortisone and ascorbic acid synergistically prevent and repair lipopolysaccharide-induced pulmonary endothelial barrier dysfunction. Chest 2017, 152, 954-962.

[s67] Egan BM, Chen G, Kelly CJ, Bouchier-Hayes DJ. Taurine attenuates LPS-induced rolling and adhesion in rat microcirculation. J Surg Res 2001, 95, 85-91.

[s68] Ergin B, Guerci P, Zafrani L, Nocken F, Kandil A, Gurel-Gurevin E, et al. Effects of N-acetylcysteine (NAC) supplementation in resuscitation fluids on renal microcirculatory oxygenation, inflammation, and function in a rat model of endotoxemia. Intensive Care Med Exp 2016, 4, 29.

[s69] Fisher BJ, Seropian IM, Kraskauskas D, Thakkar JN, Voelkel NF, Fowler AA 3rd, et al. Ascorbic acid attenuates lipopolysaccharide-induced acute lung injury. Crit Care Med 2011, 39, 1454-60.

[s70] Guarda IF, Correia CJ, Breithaupt-Faloppa AC, Gomes Ferreira S, Ramos Moreno AC, Baquerizo Martinez M, et al. Effects of ethyl pyruvate on leukocyte-endothelial interactions in the mesenteric microcirculation during early sepsis treatment. Clinics (San Paulo) 2015, 70, 508-14.

[s71] Krysztopik RJ, Bentley FR, Spain DA, Wilson MA, Garrison RN. Free radical scavenging by lazaroids improves renal blood flow during sepsis. Surgery 1996, 120, 657-62.

[s72] Krysztopik RJ, Bentley FR, Spain DA, Wilson MA, Garrison RN. Lazaroid improves intestinal blood flow in the rat during hyperdynamic bacteraemia. Br J Surg 1997, 84, 1717-21.

[s73] Lavillegrand JL, Raia L, Urbina T, Hariri G, Gabarre P, Bonny V, Bigé N, et al. Vitamin C improves microvascular reactivity and peripheral tissue perfusion in septic shock patients. Crit Care 2022, 26, 25.

[s74] Matejovic M, Krouzecky A, Rokyta R Jr, Radej J, Kralova H, Treska V, et al. Effects of combining inducible nitric oxide synthase inhibitor and radical scavenger during porcine bacteremia. Shock 2007, 27, 61-8.

[s75] Maybauer DM, Maybauer MO, Szabó C, Cox RA, Westphal M, Kiss L, et al. The peroxynitrite catalyst WW-85 improves pulmonary function in ovine septic shock. Shock 2011, 35, 148-55.

[s76] Secor D, Swarbreck S, Ellis CG, Sharpe MD, Feng Q, Tyml K. Ascorbate inhibits platelet-endothelial adhesion in an in-vitro model of sepsis via reduced endothelial surface P-selectin expression. Blood Coagul Fibrinolysis 2017, 28, 28-33.

[s77] Tyml K, Li F, Wilson JX. Delayed ascorbate bolus protects against maldistribution of microvascular blood flow in septic rat skeletal muscle. Crit Care Med 2005, 33, 1823-8.

[s78] Wang J, Song Q, Yang S, Wang H, Meng S, Huang L, et al. Effects of hydrocortisone combined with vitamin C and vitamin B1 versus hydrocortisone alone on microcirculation in septic shock patients: a pilot study. Clin Hemorheol Microcirc 2023, doi: 10.3233/CH-221444.

[s79] Wu F, Wilson JX, Tyml K. Ascorbate inhibits iNOS expression and preserves vasoconstrictor responsiveness in skeletal muscle of septic mice. Am J Physiol Regul Integr Comp Physiol, 2003, 285, R50-6.

[s80] Wu F, Wilson JX, Tyml K. Ascorbate protects against impaired arteriolar constriction in sepsis by inhibiting inducible nitric oxide synthase expression. Free Radic Biol Med 2004, 37, 1282-9.

[s81] Zhou G, Kamenos G, Pendem S, Wilson JX, Wu F. Ascorbate protects against vascular leakage in cecal ligation and puncture-induced septic peritonitis. Am J Physiol Regul Integr Comp Physiol 2012, 302, R409-16.

[s82] Sykora R, Chvojka J, Krouzecky A, Radej J, Kuncova J, Varnerova V, et al. Coupled plasma filtration adsorption in experimental peritonitis-induced septic shock. Shock 2009, 31, 473-80.

[s83] Zuccari S, Damiani E, Domizi R, Scorcella C, D’Arezzo M, Carsetti A, et al. Changes in cytokines, haemodynamics and microcirculation in patients with sepsis/septic shock undergoing continuous renal replacement therapy and blood purification with Cytosorb. Blood Purif 2020, 49, 107-113.

[s84] Alfieri A, Watson JJ, Kammerer RA, Tasab M, Progias P, Reeves K, Brown NJ, Brookes ZL. Angiopoietin-1 variant reduces LPS-induced microvascular dysfunction in a murine model of sepsis. Crit Care 2012, 16, R182.

[s85] Domizi R, Adrario E, Damiani E, Scorcella C, Carsetti A, Giaccaglia P, et al. IgM-enriched immunoglobulins (Pentaglobin) may improve the microcirculation in sepsis: a pilot randomized trial. Ann Intensive Care 2019, 9, 135.

[s86] Hoffman JN, Fertmann JM, Vollmar B, Laschke MW, Jauch KW, Menger MD. Immunoglobulin M-enriched human intravenous immunoglobulins reduce leukocyte-endothelial cell interactions and attenuate microvascular perfusion failure in normotensive endotoxemia. Shock 2008, 29, 133-9.

[s87] Kao RL, Martin CM, Xenocostas A, Huang W, Rui T. Erythropoietin improves skeletal muscle microcirculation through the activation of eNOS in a mouse sepsis model. J Trauma. 2011 Nov;71(5 Suppl 1):S462-7.

[s88] La Mura V, Pasarín M, Meireles CZ, Miquel R, Rodríguez-Vilarrupla A, Hide D, et al. Effects of simvastatin administration on rodents with lipopolysaccharide-induced liver microvascular dysfunction. Hepatology 2013, 57, 1172-81.

[s89] Landsberger M, Zhou J, Wilk S, Thaumuller C, Pavlovic D, Otto M, et al. Inhibition of lectin-like oxidized low-density lipoprotein receptor-1 reduces leukocyte adhesion within the intestinal microcirculation in experimental endotoxemia in rats. Crit Care 2010, 14, R223.

[s90] McCuskey RS, Nishida J, McDonnell D, Baker GL, Urbaschek R, Urbaschek B. Effect of immunoglobulin G on the hepatic microvascular inflammatory response during sepsis. Shock 1996, 5, 28-33.

[s91] McGown CC, Brown NJ, Hellewell PG, Reilly CS, Brookes ZL. Beneficial microvascular and anti-inflammatory effects of pravastatin during sepsis involve nitric oxide synthase III. Br J Anaesth 2010, 104, 183-90.

[s92] McGown CC, Brookes ZL, Hellewell PG, Ross JJ, Brown NJ. Atorvastatin reduces endotoxin-induced microvascular inflammation via NOSII. Naunyn Schmiedebergs Arch Pharmacol 2015, 388, 557-64.

[s93] Reis PA, Alexandre PCB, D'Avila JC, Siqueira LD, Antunes B, Estato V, et al. Statins prevent cognitive impairment after sepsis by reverting neuroinflammation, and microcirculatory/endothelial dysfunction. Brain Behav Immun 2017, 60, 293-303.

[s94] Stoyanoff TR, Rodríguez JP, Todaro JS, Colavita JPM, Torres AM, Aguirre MV. Erythropoietin attenuates LPS-induced microvascular damage in a murine model of septic acute kidney injury. Biomed Pharmacother 2018, 107, 1046-1055.

[s95] Ge W, Hu Q, Fang X, Liu J, Xu J, Hu J et al. LDK378 improves micro- and macro-circulation via alleviating STING-mediated inflammatory injury in a Sepsis rat model induced by Cecal ligation and puncture. J Inflamm (Lond) 2019, 16, 3.

[s96] Tang AL, Liu XY, Gao N, Hu TP, Yan ST, Zhang GQ. Dl-3-n-butylphthalide improves intestinal microcirculation disorders in septic rats by regulating the PI3K/AKT signaling pathway and autophagy. Int Immunopharmacol 2023, 118, 110049.

[s97] Damiani E, Ince C, Orlando F, Pierpaoli E, Cirioni O, Giacometti A, et al. Effects of the Infusion of 4% or 20% Human Serum Albumin on the Skeletal Muscle Microcirculation in Endotoxemic Rats. PLoS One 2016, 11, e0151005.
